# Evaluation of Uterine Brachytherapy as Primary Treatment Option for Elderly Patients with Medically Inoperable Endometrial Cancer—A Single-Center Experience and Review of the Literature

**DOI:** 10.3390/cancers12082301

**Published:** 2020-08-15

**Authors:** Nathalie Arians, Jan Tobias Oelmann-Avendano, Daniela Schmitt, Eva Meixner, Antje Wark, Juliane Hoerner-Rieber, Rami A. El Shafie, Kristin Lang, Markus Wallwiener, Jürgen Debus

**Affiliations:** 1 Department of Radiation Oncology, Heidelberg University Hospital, 69120 Heidelberg, Germany; jan.oelmann-avendano@med.uni-goettingen.de (J.T.O.-A.); Daniela.Schmitt@med.uni-goettingen.de (D.S.); eva.meixner@med.uni-heidelberg.de (E.M.); antje.wark@med.uni-heidelberg.de (A.W.); juliane.hoerner-rieber@med.uni-heidelberg.de (J.H.-R.); Rami.ElShafie@med.uni-heidelberg.de (R.A.E.S.); kristin.lang@med.uni-heidelberg.de (K.L.); juergen.debus@med.uni-heidelberg.de (J.D.); 2 Heidelberg Institute of Radiation Oncology (HIRO), 69120 Heidelberg, Germany; 3 National Center for Tumor diseases (NCT), 69120 Heidelberg, Germany; 4 Department of Radiation Oncology, Göttingen University Hospital, 37075 Göttingen, Germany; 5 Clinical Cooperation Unit Radiation Oncology, German Cancer Research Center (DKFZ), 69120 Heidelberg, Germany; 6 Department of Gynecology and Obstetrics, Heidelberg University Hospital, 69120 Heidelberg, Germany; markus.wallwiener@med.uni-heidelberg.de; 7 Department of Radiation Oncology, Heidelberg Ion-Beam Therapy Center (HIT), Heidelberg University Hospital, 69120 Heidelberg, Germany; 8 German Cancer Consortium (DKTK), 69120 Heidelberg, Germany

**Keywords:** endometrial cancer, elderly patients, functional inoperability, definitive radiotherapy, intrauterine brachytherapy

## Abstract

We aimed to gain more evidence regarding the feasibility, toxicity, and oncological outcome of primary brachytherapy in patients with medically inoperable endometrial cancer. Thirteen patients receiving primary brachytherapy ± external beam radiotherapy (EBRT) for endometrial cancer due to medical inoperability were identified. The Kaplan–Meier method was used to estimate overall survival (OS), progression-free survival (PFS), and local failure-free survival (LFFS). Univariate outcome analyses were performed using the log-rank test. Peri-interventional complications, acute and chronic toxicities were evaluated. Additionally, we performed a Pubmed search and review of the literature of the last 10 years. Mean age at time of diagnosis was 73.9 years (60.4–87.1 years). Eleven patients were staged FIGO IA/B and one patient each with FIGO IIIA and IIIC. Kaplan–Meier-estimated 2-/5-year LFFS were 76.2%/56.4%, respectively. High grading correlated with a worse LFFS (*p* = 0.069). Kaplan–Meier-estimated 2-/5-year PFS were 76.9%/53.8% and 2-/5-year-OS were 76.9%/69.2%, respectively. No acute toxicities > grade II and only two late toxicities grade II/III occurred. We observed three peri-interventional complications. The available evidence suggests high rates of local control after definitive brachytherapy for inoperable endometrial cancer with a favorable toxicity profile. Definitive brachytherapy +/− EBRT should be considered as the preferred approach for this patient group.

## 1. Introduction

Endometrial cancer accounts for 4.4% of all cancer cases in women, with a worldwide incidence of 382,069 in 2018 [1]. There are regional differences, with most cases being reported in highly developed countries with an annual age-adjusted incidence of 11.2 per 100,000 women (versus 3.3/100,000 in moderately/poorly developed regions) [1]. Usually, endometrial cancer develops in postmenopausal women with a mean age of about 68 years at time of diagnosis [2]. For early stages, the standard treatment of choice consists of histological confirmation of diagnosis, followed by total abdominal hysterectomy and bilateral salpingo-oophorectomy [3]. Lymphadenectomy for lymph node staging is recommended, except in very early, low-risk tumor stages [4]. Depending on the postoperative tumor stage and the presence of risk factors, postoperative radiotherapy (vaginal brachytherapy [VBT] and/or external beam radiotherapy [EBRT]) is recommended [5]. Postoperative radiotherapy has been shown to reduce the risk of locoregional recurrence, although randomized trials failed to translate this into an overall survival benefit until today. Nevertheless, there are retrospective data as well as data from population databases like the SEER (Surveillance, Epidemiology and End Results) or NCDB (National Cancer Database) database, which suggest an improved outcome for intermediate- and high-risk endometrial cancer patients after postoperative radiotherapy [6]. As a consequence of higher age and existing comorbidities, some patients diagnosed with endometrial cancer cannot be treated with surgery. Approximately ten percent of early-stage endometrial cancer patients are medically inoperable due to comorbid conditions such as cardiovascular disease, obesity-hypoventilation syndrome, diabetes-related illnesses, or a high BMI [7,8,9]. For these patients, primary radiotherapy is a potentially curative treatment option. Depending on the extent of the tumor, intrauterine brachytherapy can be applied separately or combined with EBRT. Currently, no prospective data evaluating the role of primary radiotherapy (RT) or even the role of different techniques of RT delivery in endometrial cancer patients are available. Only retrospective studies exist, which have been, to some extent, evaluated in several reviews [10]. One such study was published by the EORTC Gynecological Cancer Group in 2016 [11], recommending that RT include all regions of assumed tumor spread. Thus, only in very early stage disease with a very low risk of lymph node spread intrauterine brachytherapy should be used alone (Stage IA G1). In all other cases, intrauterine brachytherapy should be combined with EBRT to the pelvic or even para-aortal region. In any case, intrauterine brachytherapy is mandatory to achieve sufficiently high doses in the primary tumor region [11].

The aim of this retrospective study was to analyze patients of a certified oncological center regarding feasibility, toxicity, and oncological outcome, as well as to provide a review of the published literature of the last 10 years, to gain more evidence for primary radiation therapy of functionally inoperable endometrial cancer patients.

## 2. Materials and Methods

### 2.1. Patients

All in all, 1035 patients with uterine cancer presented to our gyneco-oncological center since 2009. Each year, about 90 patients (a range of 76–108 patients per year from 2009–2019) were treated, with 100 patients treated in 2019. The vast majority received surgery according to the general oncological recommendations with abdominal hysterectomy and bilateral salpingo-oophorectomy +/− lymphadenectomy. About 40–70 patients were treated with adjuvant radiotherapy (brachytherapy +/− EBRT) per year.

In general, every patient was discussed in an interdisciplinary setting to determine the most appropriate treatment concept. Only patients with localized disease and considerably high risk for anesthesia-related complications or risk of very high peri-operative morbidity as well as mortality were considered for definitive radiotherapy. As analgosedation for brachytherapy is only of short duration and the associated risks are much lower than for intubation anesthesia, most of those patients unfit for surgery were eligible for brachytherapy. In general, vaginal bleeding was also an indication for definitive radiotherapy. Applying these selection criteria, nearly all functionally inoperable patients received definitive radiotherapy. Only patients with distant metastases or with extensive disease not suitable for curatively intended radiotherapy received palliative chemotherapy (*n* = 4). Thus, the rate of patients not treated with primary surgery at our center is much below the 10% described in the literature. Hormone therapy alone was mainly applied for patients showing (inoperable) recurrence after radiotherapy and/or chemotherapy.

Using the clinical cancer registry of the National Center for Tumor Diseases (NCT), thirteen patients were identified who received primary radiotherapy for endometrial cancer because of medical inoperability (*n* = 12) or refusal of resection (*n* = 1) between 2005 and 2018 at Heidelberg University Hospital. A computerized database was used to review the medical records in order to extract patient and treatment characteristics. All data were collected retrospectively and in accordance with institutional ethical policies. The study was granted ethical approval by the local ethics committee of Heidelberg University (S-638/2019).

The following clinical data were collected: age, histology, grading, tumor stage including lymph node status according to the TNM and FIGO classification, date of first diagnosis, performance of brachytherapy including dose, applied applicator, use of additional external beam radiation therapy (EBRT), time to recurrence, pattern of recurrence, onset and localization of distant metastases, date of death, toxicities according to the National Cancer Institute (NCI) Common Terminology Criteria for Adverse Events (CTC AE) v5.0, and treatment-associated complications.

Additionally, we performed a PubMed database search of the last 10 years using the following search terms: ((medically inoperable) OR (inoperable)) AND ((endometrial) OR (endometrium)) AND ((adenocarcinoma) OR (carcinoma) OR (cancer) OR (neoplasm)) AND ((radiation) OR (irradiation) OR (brachytherapy)). Reviews and database analyses were excluded. Studies were limited to publications from the past 10 years (2010–2020). References to articles within were added if relevant (*n* = 1). Thus, we identified 36 publications. For inclusion in the review, studies should: (1) have a prospective or retrospective design; (2) include inoperable patients; (3) report outcomes on patients treated with radiation therapy as the primary treatment for endometrial cancer; and (4) regard local control, distant control, cancer-specific survival and/or overall survival as outcomes of interest. After applying these selection criteria, 15 studies remained for inclusion in the review. Data extracted from each study were as follows: the first author’s last name, year of publication, study sample size (number of patients), median or mean age, median follow-up, tumor stage, therapy type, reported local control, disease-free (DFS) or progression-free survival (PFS), cancer-specific (CSS) or disease-specific survival (DSS), overall survival (OS), and acute/late toxicities/complications.

### 2.2. Brachytherapy Treatment Methods

The brachytherapy applicator was implanted under analgosedation. Routinely, the Rotte applicator was used, if feasible. Alternatively, individual flexible catheters, the ring applicator, or a colpostate with intrauterine tube were applied. A treatment-planning CT scan using a 64-slice CT scanner with uniform slice thickness of 1.5/3.0 mm was performed with the patient in supine position. Treatment planning was performed using PLATO (Nucletron, Veenendaal, The Netherlands) until 2010 and Oncentra Brachy (Nucletron, now Elekta AB, Stockholm, Sweden) afterwards. The entire uterus and cervix were contoured as the clinical target volume (CTV). Organs-at-risks (OARs) were delineated, including the rectum and bladder, as well as the sigmoid and bowel, if required. An HDR-brachytherapy afterloading system with Iridium-192 (microSelectron, Nucletron, now Elekta until 2017 and Flexitron, Elekta afterwards) was utilized for treatment delivery. Dose was prescribed to an isodose line that covered the uterine serosa and cervix.

### 2.3. External Beam Radiation Therapy Methods

Six patients were treated with additional EBRT. EBRT was delivered to the whole pelvis using IMRT (intensity-modulated radiotherapy) techniques such as VMAT (volumetric arc therapy) (*n* = 4) or step and shoot IMRT (*n* = 1). One patient received conventional 3D pelvic radiotherapy. One patient received combination treatment of 3D and VMAT pelvic radiotherapy. EBRT to a nominal dose ranging from 45 to 56.5 Gy was applied. Contoured nodal regions included the obturator, internal and external iliac nodes, and common iliac nodal areas to the bifurcation of the aorta.

### 2.4. Statistical Analysis

Overall survival (OS), progression-free survival (PFS), and local failure-free survival (LFFS) were evaluated. Statistical events were defined as death from any cause (OS), any disease progression or death (PFS), and local failure, such as persisting tumor and tumor recurrence (LFFS). Time-to-event data were measured from the date of first diagnosis. All patients with no event at the last follow-up were censored. The Kaplan–Meier method was used to estimate LFFS, PFS, and OS for various group partitions. Univariate survival analyses were performed using the log-rank test. The statistical analysis was performed using SPSS version 24 (IBM SPSS Statistics for Windows, Version 24.0. Released 2016,IBM Corp., Armonk, NY, USA). A *p* value of <0.05 was considered statistically significant.

## 3. Discussion

The mean age of the population worldwide is rising and the percentage of the population older than 65 years in particular is increasing. Thus, the number of elderly patients diagnosed with endometrial cancer with medical comorbidities unsuitable for primary surgery will also be growing. Additionally, the effect of prosperity in society can already be noticed, as the number of patients with a high BMI is also increasing. A high BMI poses two problems: it is known to be associated with a higher risk for developing endometrial cancer, additionally, it may be a reason for functional inoperability.

The mean age of our cohort was 73.9 years, which is even far beyond the assumed age peak of 68 years for developing endometrial cancer [2]. Additionally, all patients had several comorbidities, such as cardiac, renal, or diabetes-related diseases or even prior malignancies. Thus, the risk of dying from causes other than endometrial cancer should not be underestimated in this patient group. In our cohort, seven patients died in the course of the follow-up. However, only four of them (57%) died because of disease progression.

Considering the progress that has been made in radiotherapy techniques, we performed a review of the literature of the last 10 years (2010–2020), to gain more evidence for primary radiotherapy of medically inoperable endometrial cancer patients (Table 1).

As yet, no prospective data regarding primary radiotherapy in endometrial cancer patients are available. In the last 10 years, only retrospective data of single institutions with small patient collectives or database analyses [12,13,14] have been published. Brachytherapy is the most commonly used irradiation technique for primary treatment of endometrial cancer. Local control rates and overall survival of patients treated with brachytherapy +/− EBRT vary according to the different studies. In general, age, histology, grading, tumor stage, and BMI are reported to have significant impact on oncological outcome [9,15,16,17]. In our study, 2-year OS was 76.9%. This finding is consistent with other reports in the literature. Acharya et al. [17] described a cohort (*n* = 43) of endometrial cancer patients with FIGO stages I–III, who received definitive radiotherapy because of a high BMI, rendering them functionally inoperable. The 2-year OS in that cohort was 65.2%. The 2-year cumulative incidence of pelvic failures was 8.3%, which is slightly lower than in our cohort with a 2-year-LFFS of 76.2%. However, our number also includes two patients with persisting tumor after definitive radiotherapy and, thus, a worse prognosis. Furthermore, grade 3 disease was reported to be associated with a higher risk of disease failure [17]. We could also show that patients with a grading of G3 (23%) showed a worse LFFS, although these results were not statistically significant. One explanation for this finding might be that our cohort is much smaller than that of Acharya et al. Another study by Wegner et al. [18] reported similar results of disease-specific survival with 73% at 3 years. The 1- and 2-year OS was 89% and 28%, respectively, which is much lower than in other cohorts. This study included 26 patients with early and advanced stages (FIGO stage I-III). The median age of the treated patients was quite high at 83 years, and all patients had significant medical comorbidities, which might explain the low 2-year OS. Other authors reported better outcomes than shown in our own data. For example, Nguyen et al. [19] described a 3-year uterine control and disease-free survival of 88% and 85%, respectively, in a cohort of 36 patients. Kucera et al. [20] reported a 5-year disease-specific survival of 85.4% in a cohort of 228 patients with stage I endometrial carcinoma. Furthermore, they showed that the rates of local control were related to the size of the uterus. As these patients received brachytherapy only, the size of the uterus might correlate with the dose distribution, with a better dose coverage for a smaller uterus. Additionally, the uterus was the most common site of relapse, with intrauterine recurrence in 17.5%, but only 0.4% extrauterine pelvic relapse [20]. Regarding these numbers, it must be considered that only early stage endometrial cancer patients were analyzed in the aforementioned studies, and that all data are retrospective.

In some patients presenting with endometrial cancer who are in poor general condition due to high age or many comorbidities, one might tend to omit any local therapy such as surgery or definitive radiotherapy due to concerns of overtreatment in a population with competing causes of death. However, there are data showing a benefit for local radiotherapy. For example, Staples et al. [28] could show that initial response to treatment in the form of complete or partial remission was much better after local irradiation than after hormone therapy alone. Furthermore, a review by Dutta et al. from 2017 [10] showed that any radiotherapy is associated with a benefit, compared to no local therapy in elderly patients with inoperable endometrial cancer. Brachytherapy-containing techniques provided the highest benefit to OS, but only half of all patients receiving radiation therapy also received brachytherapy. In an analysis of the National Cancer Database (NCDB), Gill et al. [14] found that brachytherapy was less likely to be delivered in elderly patients, despite the fact that omission of brachytherapy was associated with a higher likelihood of death, even in stage I endometrial cancer patients [14]. Additionally, an analysis of the SEER database by Acharya et al. confirmed this assumption, and showed that brachytherapy was associated with improved overall survival [12]. The EORTC Gynecological Cancer Group stated in a review from 2016 that intrauterine brachytherapy is the key component of definitive radiotherapy, as it is mandatory to achieve sufficiently high doses in the primary tumor region [11]. However, exclusive use of intrauterine brachytherapy should be reserved for treatment of very early stage tumors. (Stage IA G1). For example, a study be Gebhardt et al. could show very good locoregional control rates (2-y-LRC 90%) and cancer-specific survival rates (2-y-CSS 97%) for FIGO stage IA G1–2 patients receiving brachytherapy only [26]. In all other cases, intrauterine brachytherapy should be combined with EBRT. In fact, in the review by Dutta et al., too, the greatest benefit was seen for the combination of EBRT and brachytherapy. In our cohort, all patients received uterine brachytherapy, and six patients received additional EBRT. Two patients presented with more advanced stages (FIGO III), making EBRT mandatory. Furthermore, in six patients a high grading (G3) was found and in two patients type II carcinoma. These histological features are known to be associated with a higher risk of nodal spread or even distant progression. Apart from one of these patients, all others received EBRT. The cohort is too small to show any statistically significant differences between those treated with BRTH alone or a combination treatment, however. Additionally, we did not show that LFFS, PFS, or OS was associated with any radiation dose dependency.

Another option in the treatment of unresectable endometrial cancer is upfront radiotherapy, which has been evaluated in a retrospective study by Gannavarapu et al. The authors studied 29 patients who were medically inoperable, or had unresectable endometrial cancer. In that respect, this patient collective differs from other reported studies. It must be mentioned, however, that only four patients in this collective finally received surgery after chemoradiotherapy. A total of 45% had high-risk endometrial cancer. Nevertheless, 2-y-CSS was 100%, and there was no statistically significant difference between low- and high-risk endometrial cancer patients [29]. Another study also including surgically inoperable endometrial cancer patients because of local extent has been published recently by Espenel et al. [30]. They reported about 29 patients FIGO stage I-IVB receiving EBRT + brachytherapy. Oncological outcome was worse compared to other studies, with 5-y-OS of 63%, which might be due to the patient selection criteria mentioned above.

In times of rapid technological progress in the field of radiation therapy, there are only few studies evaluating other irradiation techniques than brachytherapy or conventional EBRT. Kemmerer et al. evaluated the use of stereotactic body radiotherapy (SBRT) instead of brachytherapy as a boost after EBRT in 11 patients [22]. Locoregional progression rates were quite high at 45%, and oncological outcome was relatively bad, with 18-months-OS of 57%. Furthermore, acute toxicity rates were quite high. Only one cohort described in the literature was not treated with brachytherapy or photon irradiation, but rather with carbon ions [27]. This irradiation technique is supposed to be more precise and biologically more effective than photon irradiation. Only 14 patients were included in that analysis. Despite the presumed advantages of carbon ion irradiation, oncological outcome with a 5-year LC/PFS rate of 86%/64% as well as 5-year CSS of 73% was not superior to, or was even worse than, other patient collectives receiving brachytherapy [14,21,25]. Additionally, the rates of acute and late toxicities were quite high, although only I/II° toxicities occurred.

Regarding radiation-related toxicities, the incidence of higher acute and chronic toxicities described in the literature is low. Acharya et al. reported a 4.6% incidence of acute grade III GI/GU toxicities [17]. The review by Dutta et al. described an incidence of late toxicities in the range between 0% and 21%, with only few (1.7%) grade IV late complications, which were mostly small bowel obstructions treated with surgery [10]. In our cohort, acute urogenital toxicities grade I were observed in 15.4% and grade II in 7.7%. Acute gastrointestinal toxicities grade II were described in 7.7%. The incidence of late toxicities in our cohort was 15.4%, with one case of urogenital toxicity grade III requiring urological intervention and one case of gastrointestinal toxicity grade II. No reports of grade IV late toxicities, especially no small bowel obstructions, were observed. Additionally, we observed three peri-interventional complications in the form of perforation of the uterus by the brachytherapy applicator. The risk for perforation was higher when using a flexible catheter instead of conventional brachytherapy applicators because of the unusual anatomy of the uterus. However, it must be noted that the perforation of the uterus with an individual flexible catheter did not have any negative consequences for the patient. No bleedings or infections were observed, and radiation treatment could be completed as planned. One patient died after perforation of the uterus with the Rotte applicator. In this patient, intra-abdominal bleeding was observed, which could have been arrested by surgical intervention. However, the patient refused any operation or blood transfusion for religious reasons.

The greatest weaknesses of our and many other studies are, of course, the small number of patients, the heterogeneity of the applied radiation regimens, and the retrospective nature. However, as data on definitive radiotherapy in patients with inoperable endometrial cancer are rare and prospective data are lacking completely, any available data can help us gain more evidence which might be of benefit for individual, patient-centered decision-making.

## 4. Results

### 4.1. Patient and Treatment Characteristics

Mean age at time of diagnosis was 73.9 years (60.4–87.1 years). Diagnosis of endometrial carcinoma was histologically confirmed by hysteroscopy and fractionated abrasion. Most patients were diagnosed with tumor stage FIGO IA/B (*n* = 11), one patient presented with lymph node metastases (FIGO IIIC), and one patient was diagnosed with FIGO stage IIIA. Histologically, eleven patients had type I uterine carcinoma and two had type II carcinoma. Grading was defined as follows: G1 (*n* = 4), G2 (*n* = 6), and G3 (*n* = 3) (Table 2). Reasons for inoperability were always combinations of comorbidities, such as cardiac (*n* = 8), cerebral (*n* = 2), vascular (*n* = 2), and/or pulmonal comorbidities (*n* = 5), diabetes (*n* = 6), and diabetes-related renal disease (*n* = 3), prior pelvic malignancy (*n* = 1; rectal cancer cured by extensive surgery), or high BMI (body mass index; *n* = 7).

Seven patients received brachytherapy only. In most cases, the Rotte applicator was used for intrauterine brachytherapy (*n* = 9). Other applicators used included the ring pen applicator (*n* = 1), colpostate with pen (*n* = 1), or individual flexible catheters (*n* = 2).

Six patients received additional EBRT. Two patients received a total dose of 54 Gy in 1.8 Gy per fraction (*n* = 2), three patients 45 Gy in 1.8 Gy per fraction, one of whom received an integrated boost to suspicious lymph nodes with a cumulative dose of 56.2 Gy (single dose 2.26 Gy), and one a sequential boost to the uterus up to 55.8 Gy, respectively. One patient received three fractions with 3 Gy delivered via AP/PA fields as emergency radiotherapy because of vaginal bleeding and 20 fractions VMAT with 1.8 Gy (cumulative dose 45 Gy). The different fractionation regimens are listed in Table 3.

Only one patient received additional chemotherapy as adjuvant therapy after completing definitive radiotherapy consisting of EBRT and brachytherapy for a FIGO stage IIIA tumor, with six cycles of carboplatin and paclitaxel.

### 4.2. Survival Data

Median follow-up time was 78.8 months. Local failure (persisting tumor or local recurrence) was diagnosed in five patients (5/13; 38%). Local recurrence was documented in three patients (3/13; 23%), two of whom presented with vaginal bleeding. Two patients died in the course of the follow-up. Two patients (2/13; 15%) showed persisting tumor after radiotherapy. These two patients also developed distant metastases (peritoneal, hepatic, pulmonal, and lymphatic) and died 23.2 and 23.7 months after first diagnosis, respectively.

**Figure 1:**
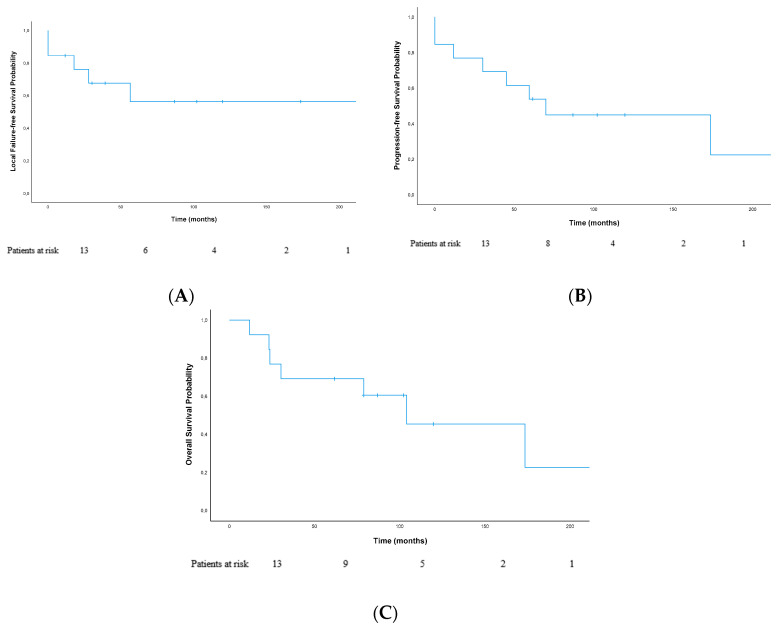
Survival analysis. Kaplan–Meier-estimated (**A**) local failure-free survival (LFFS), (**B**) progression-free survival (PFS), and (**C**) overall survival (OS) of the whole cohort.

Median LFFS (local failure-free survival) of the whole cohort was 61.6 months. Kaplan–Meier-estimated median LFFS was not reached at time of analysis. The 2- and 5-year LFFS were 76.2% and 56.4%, respectively (Figure 1A). A grading of G3 correlated with a worse LFFS, but results did not reach statistical significance (*p* = 0.069).

Median PFS (progression-free survival) of the whole cohort was 61.6 months. Kaplan–Meier-estimated median PFS was 69.8 months; 2- and 5-year PFS were 76.9% and 53.8%, respectively (Figure 1B).

In total, seven patients died. Kaplan–Meier-estimated median OS (overall survival) was 103.9 months (22.1; 185.8); estimated 2- and 5-year OS were 76.9%/69.2%, respectively (Figure 1C). OS was not significantly different in patients with local recurrence (*n* = 5) compared to patients without local recurrence (*n* = 8) (*p* = 0.99).

Statistical analyses regarding FIGO stage (FIGO I: *n* = 11, FIGO III: *n* = 2), histological type (type I: *n* = 11; type II: *n* = 2), use of EBRT (*n* = 6), or use of different brachytherapy applicators (Rotte applicator: *n* = 9; ring pen applicator: *n* = 1; colpostate with pen: *n* = 1; individual flexible catheters: *n* = 2) as well as the total dose (EQD2, alpha/beta 3) (EQD2 < 70: *n* = 1; EQD2 70–80: *n* = 3; EQD2 80–90: *n* = 3, EQD2 90–100: *n* = 3; EQD2 >100: *n* = 3 did not show any statistically significant differences in OS, PFS, or LFFS (Table 4).

### 4.3. Treatment Tolerance and Toxicity

Peri-interventional complications were observed in three patients. Two patients experienced perforation of the uterus using flexible catheters without presenting any clinical symptoms or developing further complications. Another patient died because of hemorrhagic shock after perforation of the uterus using the Rotte applicator, as she refused blood transfusions for religious reasons. All perforations were diagnosed by CT scans, which were performed for treatment planning after insertion of the applicator. One patient became hemodynamically unstable during peridural anesthesia, so that only one of two planned brachytherapy sessions could be conducted.

Acute toxicities were defined as any toxicity emerging during and up to 3 months after completing radiotherapy. The following acute toxicities were observed: bladder toxicity CTC grade I in 2/13 patients (15.4%), urinary tract infection in 2/13 patients (15.4%), stress urinary incontinence grade II in 1/13 patients (7.7%), and diarrhea grade II in 1/13 patients (7.7%). One patient (7.7%) with obesity developed epitheliolyses in the subabdominal skinfold during radiotherapy. No other skin reactions were observed.

The following late toxicities were observed: One patient (7.7%) presented with stenosis of the ureter 4 months after completing definitive radiotherapy that required urological intervention. Chronic urinary incontinence grade II was observed in one patient (7.7%). The same patient reported chronic proctitis and diarrhea CTC grade II (7.7%).

## 5. Conclusions

The available evidence suggests high rates of local control for elderly women with medically inoperable endometrial cancer after definitive brachytherapy with or without EBRT with a relatively favorable toxicity profile. Peri-interventional complications using dedicated brachytherapy applicators, such as the Rotte applicator, are rare. Caution is required when using applicators other than standard brachytherapy applicators. Definitive radiotherapy—preferably as a combination of EBRT and brachytherapy—should be considered as the preferred approach for this patient collective.

## Figures and Tables

**Table 1:** Review of published data from 2010 to 2020.

Author, Year	Recruitment Period	Patient No.	Median Follow-Up	Median Age	FIGO Stage	Treatment Type	Local Control	DFSDSS/CSS/PFS	OS	Complications/Toxicity
Inciura et al., 2010 [15]	1995–1998	29	4.6 years	75 Years (mean)	I–III	HDR-BRTH + EBRT	17.2% local failure	5-/10-y-DSS 73.5%, 67.9% *	5-/10-y-OS 48.3%/20.7% **	7% acute toxicities I°, 13.8% late complications I-II°
Wegner et al., 2010 [18]	1997–2008	26	12 months	83 years	I–III	73% HDR-BRTH + EBRT, 27% HDR-BRTH only	1-/2-y-LC 100%/75%	1-/3-y-DSS 93%/73%	1-/2-y- OS 89%/28%	8% late complications
Ohkubo et al., 2011 [21]	2002–2006	10	55 months	72 years	I–II	HDR-BRTH + EBRT (*n* = 9), HDR-BRTH only (*n* = 1)	5-y-LC 100%	No cancer specific deaths	5-y-OS 90%	70% acute I-II° toxicities; 30% late I° toxicities
Podzielinski et al., 2012 [9]	1997–2009	74	31 months	65 years	I–II	79% BRTH + EBRT, 17% BRTH alone (LDR- or HDR-BRTH), 4% EBRT alone		Median PFS 43.5 months, 3-y-PFS 68%. Median time to death following recurrence: 13.7 months	Median OS 47.2 months ***	8.1% acute III° toxicities, 4% IV° AEs
Kemmerer et al., 2013 [22]	2006–2011	11	10 months	78 years	I + III	EBRT + SBRT boost	45% locoregional progression	12-/18-months FFP 68%/41%; (18-months-FFP:100% for stage IA, 33% for stage IB; 100% for G1)	18-months-OS 57%	73%/9% acute I°/III°gastrointestinal toxicities, 18% acute genitourinary toxicities I°, 18% I° and II° skin toxicity, respectively. No late toxicities.
Zhou et al., 2015 [23]	2007–2011	31	54.8 months	55.9 years	I–III	^252^Californium Neutron BRTH +/− EBRT	LCR Stage I: 100% Stage II: 81.8% Stage III: 50% All: 80.6%	DSS Stage I: 100% Stage II: 54.5% Stage III: 0% All: 54.8%	5-y-OS Stage I: 80% Stage II 54.5% Stage III 0% All: 51.6%	12.9% late toxicity II°
Gill et al., 2014 [24]	2007–2013	38	15 months	69 years	I	HDR-BRTH + EBRT (*n* = 18), HDR-BRTH only (*n* = 20)	2-y-LC 90.6%	No regional or distant metastases	2-y-OS 94.4%	1 acute bleeding requiring transfusion, no other > II° acute toxicities. No II-V° late toxicities.
Acharya et al., 2016 [17]	2003–2015	43	29.7 months	62 years	I–III	HDR-BRTH + EBRT (*n* = 15), HDR-BRTH only (*n* = 28),	2-y-incidence of pelvic failure 8.3%/	2-y-incidence of distant failure 13.5%****	2-y-OS 65.2% (BRTH alone 69.4% vs. BRTH + EBRT 57.9%)	53%/4.7% I-II°/III° acute toxicities; 4.7% late toxicities including one recto-vaginal fistula IV°. 1 uterine fundus perforation.
Jordan et al., 2017 [25]	2010–2016	15	57 months	69.3 years	I–II	HDR-BRTH + EBRT (*n* = 8), HDR-BRTH only (*n* = 7)	93.4% at 4 years			8% of patients with at least one side effect, no > II° toxicities
Draghini et al., 2017 [16]	2005–2016	17	53 months	79 years	I–III	HDR-BRTH + EBRT (*n* = 3), HDR-BRTH only (*n* = 14),	3-y-/6-y-LC 86%/69%*****	1-/2-/6-y-CSS 93%/85%/85%		12% acute toxicities II°, 12% late toxicities I°
Gebhardt et al., 2017 [26]	2007–2016	45	18.6 months	63 years	IA G1–2	HDR-IGBT	2-y-locoregional control 90%	2-y-cancer-specific survival 97%	2-y-OS 86%	No acute toxicities > II°, no late toxicities
Irie et al., 2018 [27]	1998–2014	14	50 months	70 years	I–III	C12-RT (NO BRTH)	5-y-LC 86%	5y-PFS 64%, 5-y-CSS 73%	5-y-OS 86%	8 acute toxicities I/II°, 14 late toxicities I/II°
Staples et al., 2018 [28]	2000–2016	51	20.5 months	66 years	I, II	Hormone therapy (45.1%), RT [(49%), 40% BRTH alone, 56% BRTH + EBRT, 4% EBRT alone] or a combination (5.9%) ^#^	Initial CR/PR: 38.1% (Hormone therapy), 63.6% (RT), 100% (combination group)	-	-	In case of salvage hysterectomy: 12.5% peri-operative mortality
Gannavarapu et al., 2020 [29]	2012–2019	29	17 months	59 years	I–III	HDR-BRTH + EBRT (*n* = 22), HDR-BRTH only (*n* = 7), CAVE: 5 patients received surgery	2 local recurrences	3 distant recurrenecs in the HREC group; 2-y-cumulative recurrence 44% (HREC) and 7% (LREC); 2-y-CSS 100%	2-y-OS 73% (HREC) and 77% (LREC)	no acute toxicities ≥/= III°; 1 late toxicity IV° (cystitis), 1 late toxicity III° (rectal bleeding)
Espenel et al., 2020 [30]	2002–2017	27	36.5 months	70.4 years	I–IVB	EBRT + 3D image-guided BRTH	cumulative incidence of local/pelvic failures 19%/7%	Cumulative incidence of distant failures 26%	5-y-OS 63%	15% late urinary and 7% gastro intestinal toxicities ≥ II°; No vaginal toxicity ≥ II°.

* significant differences depending on stage, histology, and grading; ** significant differences depending on stage; *** significant differences depending on age, grading, and BMI; **** G3 predicted for a higher risk of all-failures; ***** significant differences depending on histology; y = year, AE = adverse event, CR = complete remission, PR = partial remission, DFS = disease-free survival, DSS = disease-specific survival, PFS = progression-free survival, HDR-IGBT = high-dose-rate image-guided brachytherapy, C12-RT = carbon-ion radiotherapy, SBRT = stereotactic body radiation therapy, FFP = freedom from progression, HREC = high-risk endometrial cancer, LREC = low-risk endometrial cancer, ° = grade.

**Table 2:** Histopathological characteristics.

Histopathological Characteristics	*n*
Stage (FIGO)	
I	11
IA	5
IB	3
IIIA	1
IIIC	1
Grading	
G1	4
G2	6
G3	3
Histology	
endometrioid	8
serous	2
mucinous	1
tubular-papillary	1
unknown	1

FIGO = Fédération Internationale de Gynécologie et d’Obstétrique.

**Table 3:** Fractionation regimens.

Pat.	EBRT			BRTH			Cum. EQD2
	Cum. Dose	Single Dose	EQD2	Single Dose	Fractions	EQD2	
1	50.4	1.80	48.38	8	3	52.8	101.18
2	45/55.8	1.80	53.57	8	1 (2 were planned)	17.6	71.17
3	-	-	-	7	5	70	70
4	-	-	-	8	4	70.4	70.4
5	50.4	1.80	48.38	8	3	52.8	101.18
6	-	-	-	8 + 5	3 + 2	68.8	68.8
7	45	1.8	43.2	8	3	52.8	96
8	-	-	-	8	5	88	88
9	45	3 (3 Fx)/1.8 (20 Fx)	45.36	8	3	52.8	98.16
10	45/56.5	1.8/2.26	59.44	7	3	42	101.44
11	-	-	-	8	5	88	88
12	-	-	-	8.5	5	97.75	97.75
13	-	-	-	8	5	88	88

All dose specifications are in Gray (Gy); EBRT = external beam radiotherapy, BRTH = brachytherapy, EQD2 = biologically equivalent dose for 2 Gy, cum = cumulative, Fx = fraction, Pat. = patient.

**Table 4:** Survival time comparisons of different group partitions.

	LFFS		PFS		OS	
	Chi-Squared	*p*-Value	Chi-Squared	*p*-Value	Chi-Squared	*p*-Value
FIGO stage	0.175	0.676	0.013	0.91	0.12	0.729
Histological type	0.175	0.676	0.013	0.91	0.24	0.624
Grading	5.35	0.069	1.585	0.453	1.743	0.418
EBRT	0.191	0.662	0.014	0.904	0.31	0.577
Applicator type	1.662	0.645	1.572	0.666	2.586	0.46
EQD2	1.475	0.831	1.536	0.82	0.644	0.958

Univariate survival time comparisons were performed using the log-rank test. LFFS = local failure-free survival, PFS = progression-free survival, OS = overall survival, FIGO = Fédération Internationale de Gynécologie et d’Obstétrique, EBRT = external beam radiation therapy, EQD2 = biological equivalent dose for 2 Gray.
